# How to do genetic counseling in psychiatry?

**DOI:** 10.1192/j.eurpsy.2021.158

**Published:** 2021-08-13

**Authors:** M. Johansson Soller, R. Moldovan, C. Ingvoldstad Malmgren, A. Cuthbert, M. Rietschel

**Affiliations:** Clinical Genetics, Karolinska Hospital/Region Stockholm, Stockholm, Sweden

**Keywords:** Genetic, Counselling, schizophrénia, bipolar

## Abstract

Genetic counselling has been defined as the process of helping people “understand and adapt to medical, psychosocial, and familial aspects of genetic conditions.” It can also help patients and families deal with stigma and understand the significance of possible genetic findings. Psychiatric genetic counselling (PGC) is an emerging field aimed to help people with a personal or family history of psychiatric illnesses such as schizophrenia, bipolar disorder, or neuropsychiatric conditions, to understand genetic etiological mechanisms as a critical component. Counselling strategies are used to identify and adapt to psychological and familial consequences of the conditions and to reduce stigma surrounding the psychiatric illness. A recent survey showed that PGC is still not routinely offered and usually only discussed at the initiative of the patient, e.g. if they ask about the possibility of “hereditary" illness, or if a caregiver during a session for another indication, identifies the family history. If a monogenetic or chromosomal cause is identified, the genetic counselling follows a more traditional path, but if, on the other hand, the cause is complex, the counselling will not be as clearcut. It will then focus on explaining risk for disease with quite uncertain riskscores as no causative genetic change is identified. Although genetic testing most often cannot be offered and individual risk scores based on genetic markers cannot be given, there is still great value for patients and their relatives in PGC. Studies have shown that the effect of PGC is an increase of empowerment and a reduction of stigma.

**Disclosure:**

No significant relationships.

